# Neutrophil L-Plastin Controls Ocular Paucibacteriality and Susceptibility to Keratitis

**DOI:** 10.3389/fimmu.2020.00547

**Published:** 2020-04-03

**Authors:** Xiaoxiao Lu, Abirami Kugadas, Kirsten Smith-Page, Jeffrey Lamb, Tiffany Lin, Yusha Ru, Sharon Celeste Morley, Raina Fichorova, Sharad K. Mittal, Sunil K. Chauhan, Sejiro Littleton, Daniel Saban, Mihaela Gadjeva

**Affiliations:** ^1^Division of Infectious Diseases, Department of Medicine, Brigham and Women's Hospital and Harvard Medical School, Boston, MA, United States; ^2^Washington University School of Medicine, St. Louis, MO, United States; ^3^Laboratory of Genital Tract Biology, Department of Obstetrics, Gynecology and Reproductive Biology, Brigham and Women's Hospital and Harvard Medical School, MA, United States; ^4^Schepens Eye Research Institute, Massachusetts Eye & Ear Infirmary and Harvard Medical School, Boston, MA, United States; ^5^Duke Department of Ophthalmology, Duke Eye Center, Durham, NC, United States

**Keywords:** commensals, neutrophils, keratritis, *P. aeruginosa*, macrophages, infection

## Abstract

Why ocular mucosa is paucibacterial is unknown. Many different mechanisms have been suggested but the comprehensive experimental studies are sparse. We found that a deficiency in L-plastin (LCP1), an actin bundling protein, resulted in an ocular commensal overgrowth, characterized with increased presence of conjunctival *Streptococcal* spp. The commensal overgrowth correlated with susceptibility to *P. aeruginosa*-induced keratitis. L-plastin knock-out (KO) mice displayed elevated bacterial burden in the *P. aeruginosa*-infected corneas, altered inflammatory responses, and compromised bactericidal activity. Mice with ablation of LPL under the LysM Cre (*LysM. Cre*^*pos*^*LPL*^*fl*/*fl*^) and S100A8 Cre (*S100A8.Cre*^*pos*^*LPL*^*fl*/*fl*^) promoters had a similar phenotype to the LPL KOs mice. In contrast, infected *CD11c.Cre*^*pos*^*LPL*^*fl*/*fl*^ mice did not display elevated susceptibility to infection, implicating the myeloid L-plastin-sufficient cells (e.g., macrophages and neutrophils) in maintaining ocular homeostasis. Mechanistically, the elevated commensal burden and the susceptibility to infection were linked to defects in neutrophil frequencies at steady state and during infection and compromised bactericidal activities upon priming. Macrophage exposure to commensal organisms primed neutrophil responses to *P. aeruginosa*, augmenting PMN bactericidal capacity in an L-plastin dependent manner. Cumulatively, our data highlight the importance of neutrophils in controlling ocular paucibacteriality, reveal molecular and cellular events involved in the process, and suggest a link between commensal exposure and resistance to infection.

## Introduction

L-plastin (LPL) is a leukocyte-specific member of the plastin family of actin remodeling proteins. In humans, there are two ubiquitous plastin isoforms (L and T). Expression of the L isoform has been reported exclusively in the hemopoietic cell lineages, while the T isoform has been found in all other normal cells of solid tissues that have replicative potential (fibroblasts, endothelial cells, epithelial cells, melanocytes, etc.) (1, 2). Zebrafish, murine, and human L-plastin share over 85% aminoacid sequence identity suggestive that their function is highly evolutionary conserved and important (3).

L-plastin knock outs have multiple defects in many cell types. Because of L-plastin ability to support actin fibers, it regulates immunological synapse formation in B- and T-cells, their motility, and functions, such as antibody responses (4–6). In addition, several reports document that L-plastin controls innate immunity. L-plastin deficient zebrafish show increased susceptibility to lung bacterial opportunistic infections at the time of zebrafish development when the adaptive immunity hasn't matured, implicating defects in innate immunity (3). Similarly, L-plastin deficient mice show elevated susceptibility to pneumococcal infections (7). The phenotype is likely due to impaired generation of CD11c^+^ alveolar macrophages in the lungs since L-plastin deficient and the conditionally floxed LPL (*CD11c.Cre*^*pos*^*-LPL*^*fl*/*fl*^) mice have decreased numbers of CD11c^+^ macrophages at baseline and during infection (7). Impaired localization of macrophage precursors to the alveoli is reported in LPL deficient mice (8). While both LPL KO mice and *CD11c.Cre*^*pos*^*-LPL*^*fl*/^^fl^ mice have defects in handling of pneumococci, it remains unclear whether the lower numbers of LPL-deficient alveolar macrophages or other myeloid defects are causative for the increased susceptibility to disease. To this end, defective responses are detected in L-plastin deficient polymorphonuclear cells (PMNs). Namely, L-plastin-deficient PMNs show impaired killing of *Staphylococcus aureus* and *E. coli* (9, 10). Cumulatively, these studies provide a solid foundation for further work that should elucidate which L-plastin regulated pathways and which myeloid cell types sensitize to infection. The generation of the floxed-L-plastin mouse strains offer tools to address the issue.

Our interest in L-plastin-regulated biology came from the discovery that the relative abundances of the L-plastin-derived peptides were significantly elevated in the proteomes from eye wash samples derived from specific pathogen free (SPF) mice when compared to germ free mice (GF) (11). In addition, our data indicated that the commensal presence promoted relative abundances of innate immune molecules with antimicrobial activities and recruitment of neutrophils. Based on these data we suspected an important role for L-plastin in responding to commensal organisms and/or pathogens. Since we reasoned that there was a connection between responses to commensal organism and susceptibility to infection, we were interested in understanding what role L-plastin plays in this process and which cell types were affected.

Here, we report that *LPL KO* mice had conjunctival commensal overgrowth exemplified by increased levels of *Streptococcus ovis* (*S. ovis*) and exhibited profound susceptibility to *Pseudomonas aeruginosa*-induced keratitis. Infected mice with specific ablation of L-plastin under the *LysM. Cre*^*pos*^ and *S100A8/A9 Cre*^*pos*^ promoters showed a similar phenotype to the LPL KO mice, signifying the importance of myeloid cells and, specifically, neutrophils in mediating the phenotype. In contrast, the *CD11c.Cre*^*pos*^*-LPL*^*fl*/*fl*^ mice did not display increased susceptibility to infection. Mechanistically, L-plastin deficient neutrophils responded poorly to priming signals released by trained macrophages.

## Materials and Methods

### Ethics Statement

All animal experiments were performed following National Institutes of Health guidelines for housing and care of laboratory animals and performed in accordance with institutional regulations after protocol review and approval by the BWH Animal Care and Use Committee and were consistent with the Association for Research in Vision and Ophthalmology guidelines for studies in animals. Additional experiments were performed and reviewed by BWH Animal Care and review Committee.

### Mice

Mice were housed and bred in the Channing Laboratory Animal Care Facilities. Age and gender matched L-plastin KO mice and WT littermates at 7–9 weeks old, gender-matched, were used throughout the experiments. The majority of the experiments were carried out with female mice as the initial experiments showed no gender bias (Supplementary Figure 2).

### Genotyping

Breeders of LPL KO, wild type littermates, LPL floxed, and CD11c.Cre^pos^-LPL^fl/fl^ mice were generously provided by Dr. Morley, Washington University School of Medicine, St. Louis, MO (8, 12, 13). Genotyping of the LPL KO, wildtype, CD11c.Cre^pos^-LPL^fl/fl^ mice was performed as previously described using the primers: LPL mutant: ATCGCCTTCTATCGCCTTCTTG; LPL Forward: GCTCCATCATTTCTTCGTCAG; LPL Reverse: TCACCTCCTTCCTTCATCCTTG; Cre 1: AGG TTC GTT CAC TCA TGG A; Cre 2: TCG ACC AGT TTA GTT ACC C; IntC500F: CCT CCG GAG AGC AGC GAT TAA AAG TGT CAG; IntC500R: TAG AGC TTT GCC ACA TCA CAG GTC ATT CAG; LCP-geno-For2: AAGGATTGCAGAAGCAGGTAGGGCT; LCP-geno-Rev2: GGGCATATGTACATGTAGAGGTCACA. *LysM Cre*^*pos*^*-LPL*^*fl*/*fl*^ and *S100A8.Cre*^*pos*^*-LPL*^*fl*/*fl*^ mice were generated by crossing floxed LPL^fl/fl^ alleles onto LysM Cre (Jackson) and MRP8-Cre-ires/GFP, Mrp8 cre^Tg^ (Jackson). The genotyping of those strains was carried out per vendor's instructions.

### Bacterial Strains and Inocula

Invasive *P. aeruginosa* strains 6294 and PAO1 were used throughout these experiments. The bacterial strains were grown overnight at 37°C on Tryptic Soy Broth (TSB) (Cardinal Health) agar plates supplemented with 5% sheep blood. The bacterial suspensions were prepared in saline solution and used for subsequent infection experiments.

### Infection Model

Infections were carried out as described previously (14). Briefly, mice were anesthetized with intraperitoneal ketamine and xylazine injections. Three 0.5 cm scratches were made on the cornea with 25G needle tip and an inoculum of 5 × 10^5^ cfu of *P. aeruginosa 6294* or 5 × 10^6^ cfu of *P. aeruginosa PAO1* delivered in 5 μl onto the eye. Mice remained sedated for ~30 min. For evaluation of corneal pathology, daily scores were recorded by an observer unaware of the experimental status of the animals based on the following scoring system using a graded scale of 0 to 4 as follows: 0, eye macroscopically identical to the uninfected contra-lateral control eye; 1, faint opacity partially covering the pupil; 2, dense opacity covering the pupil; 3, dense opacity covering the entire anterior segment; and 4, perforation of the cornea, phthisis bulbi (shrinkage of the globe after inflammatory disease), or both. To determine corneal bacterial counts at 24 h after infection, mice were sacrificed, the eyes were enucleated, and the corneas were dissected from the ocular surface. To quantify *P. aeruginosa* levels, corneas were suspended in PBS, 0.05% Triton X100, serially diluted and plated on *P. aeruginosa* selective McConkey agar plates.

### Histopathology Examinations

Eyes were enucleated from euthanized mice, fixed in 4% (v/v) paraformaldehyde, and subsequently embedded in paraffin. Four micrometer sections were cut and stained with hematoxylin-eosin to visualize tissue morphology following previously used techniques (15). The levels of ocular inflammation in the corneal sections was quantified on a scale of 1 to 4, with “1” being reflective of no neutrophil influx in the cornea or anterior chamber and healthy appearance; “2” denoting mild inflammation, preserved corneal epithelial layer, presence of neutrophils in the conjunctival tissues; “3” being reflective of moderate inflammation, loss of epithelial layer, influx of neutrophils in the corneal epithelium, less than 50 cells/field of vision at 40X magnification, neutrophils lining the anterior chamber; and “4” denoting severe inflammation, lost corneal epithelial layer, massive influx of neutrophils in the cornea (more than 50 cells/field of vision at 40X magnification); numerous neutrophils present and scattered thought the anterior chamber. Histological scoring was carried out by Dr. Roderick Bronson, (HMS, Histopathology core) blindly using sections which did not display genotypic and phenotypic information.

### Cytokine Analysis

Cytokine levels were determined by commercially available ELISA assays (R&D Systems).

### Single Cell Suspensions of Cornea and Conjunctiva Tissues, and Bone Marrow

Single cell suspension of cornea and conjunctiva were made according to the method described by Khandelwal et al. (16) with the following change. Tissues were cut into small pieces and were minced and digested with collagenase D (2 mg/ml) (Roche, Germany) for 1 h with vortexing every 15 min. Bone marrow were flushed and filtered through 70 μM filter. Single cell suspensions were counted and used for FACS analysis.

### Flow Cytometry

All the cells were incubated with Fc block (Biolegend) for 15 min at room temperature before staining for specific markers. Two million corneal and conjunctival cells were stained for 30 min with CD45 FITC, LY6G APC, CD11b PE (Biolegend). Bone marrow cells were stained with the following panel of antibodies CD3e BUV395, CD19 BV650, c-Kit BV711, CD11b FITC, Ly6G PE, Ter119 PECy7, Ly6C APC, F4/80 APC/Cy7 (BD Biosciences). Appropriate isotypes were used as negative controls. Stained cells were washed with PBS and analyzed on LSR-II flow cytometer (BD Biosciences). The data acquired from LSR-II were analyzed by Summit.

### Purification of PMNs and Bactericidal Assays

Murine bone marrow was flushed from both hind limbs with PBS supplemented with 2% fetal bovine serum and 1 mM EDTA. The cells were washed, erythrocytes in the cell pellet were lyzed using the Mouse Erythrolysis Kit (R&D Systems) according to the manufacturer's instructions, and neutrophils were isolated using the EasySep Mouse Neutrophil Enrichment Kit (Vancouver, Canada). Neutrophils were incubated with *P. aeruginosa* strain PA01 at an MOI of 100:1 for 90 min at 37°C on a rotator. Aliquots taken at time 0 and 90 min were serially diluted and plated on McConkey agar to determine numbers of live *P. aeruginosa*. Percentage of killing ability of neutrophils was calculated as in Dwyer and Gadjeva (17).

### Measurement of Reactive Oxygen Species

Two million bone marrow purified neutrophils were incubated with 50 μl of 50 μM luminol sodium salt (Sigma, A4685) and 5 μl of peroxidase from horseradish (1 μ/ul) (Sigma, P8375) in a total of 200 μl reaction in HBSS^+/+^ at 37°C for 5 min. *Pseudomonas aeruginosa* 6294 was spiked at an MOI of 1 or 5 in 10 μl HBSS^+/+^ just before recording the luminescence. Luminescence was measured every 1 min for a total of 30 min duration in SpectraMax L (Molecular Devices) at 470 nm wave length.

### Gut Microbiome Profiling by 16S rRNA Sequencing

DNA was extracted from the fecal pellets using QIAamp DNA Stool Mini Kit (Qiagen). Quality of the DNA was checked by Agilent 2100 Bioanalyser. Libraries were created by targeting the V4 region of the 16S rRNA gene using qPCR. Purified and size selected libraries were subjected for sequencing by Illumina MiSeq. The sequencing was performed at SeqMatic (Fremont, CA).

Sequence analysis was carried out using Pavian R package 0.8.2.

### Bacterial Identification

The identification of the commensal organisms was carried out at the BWH Microbiology Core facility using Vitek® MS.

### Statistical Analysis

Statistical analysis of corneal pathology scores, bacterial burden, and cytokine levels were either by Mann-Whitney U-test for pair-wise comparisons or the Kruskal-Wallis non-parametric ANOVA with Dunn's correction for Multigroup comparisons and individual 2-group comparisons (Prism 4.0 for Macintosh). Differences were considered significant if the *p* < 0.05 (Prism 4.0 for Macintosh).

## Results

### L-Plastin Deficiency Sensitizes to *P. aeruginosa*-Induced Keratitis

**Figure 1:**
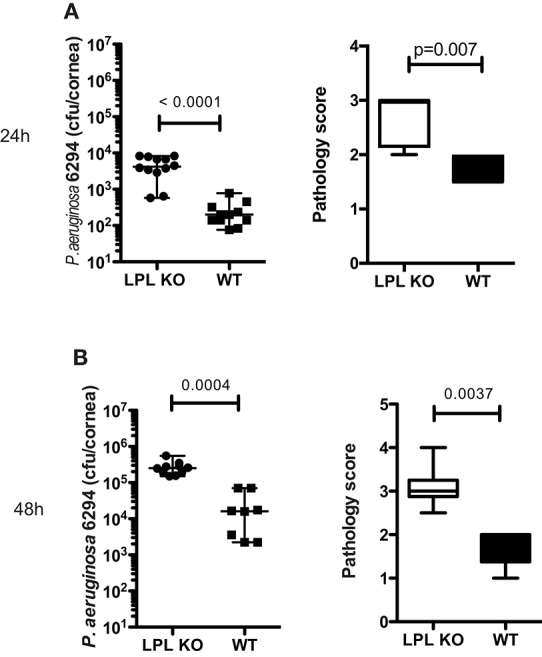
L-plastin deficiency sensitizes to *P. aeruginosa*-induced keratitis. **(A)** At 24 h post-infectious challenge with *P. aeruginosa* 6294. Groups of LPL KO mice (*n* = 12) and WT (*n* = 11) mice were infected with 5 × 10^5^ CFU *P. aeruginosa* 6294 per eye. Data are representative of three independent experiments performed under comparable conditions. *p*-values are generated using Student's *t*-test, *p* = 0.0001 Pathology scores at 24 post-infection. *p*-values are generated using Mann-Whitney test, *p* = 0.007. **(B)** At 48 h post-infectious challenge with *P. aeruginosa* 6294. Groups of LPL KO mice (*n* = 9) and WT (*n* = 8) mice were infected with 5 × 10^5^ CFU *P. aeruginosa* 6294 per eye. Data are representative of three independent experiments performed under comparable conditions. *P*-values are generated using Student's *t*-test, *p* = 0.0004. Pathology scores at 48 post-infection. *P*-values are generated using Mann-Whitney test, *p* = 0.0037. Cumulatively these data show significant susceptibility to *P. aeruginosa*-induced infection in the absence of L-plastin.

To determine the impact of L-plastin deficiency on *P. aeruginosa*–induced keratitis, L-plastin KO mice and wild type (WT) littermates were infected with *P. aeruginosa* strain 6294. Elevated bacterial counts were detected in the infected LPL KO mice when compared to the WT littermates at 24 and 48 h post-infection (Figures 1A,B, *p* = 0.0001, *p* = 0.004, Student's *t*-test). The LPL KO mice persistently had elevated corneal opacity, demonstrating a stable trend for worse disease (Figures 1A,B, *p* = 0.007 and *p* = 0.0037, Mann-Whitney). To rule out bacterial strain-specific responses, additional infection experiments were carried out with the laboratory strain, *P. aeruginosa* PAO1, and a similar tendency for elevated susceptibility to infection was observed. At 24 h after infection the corneas of the infected LPL KO mice exhibited a significantly higher bacterial burden (Supplementary Figure 1A, *p* < 0.0001, Student's *t*-test) than those in the control littermates (Supplementary Figure 1A). Initially, there were lower pathology scores in the PAO1 infected LPL KO mice when compared to the WT littermates (Supplementary Figure 1A, *p* = 0.0062, Mann-Whitney). However, further monitoring of disease progression revealed a sustained tendency for worse disease in the LPL KO (Supplementary Figure 1B). Bacterial burdens were significantly increased in the infected corneas from LPL KO mice at 48 h post-challenge (Supplementary Figure 1B, Student's *t*-test, *p* = 0.03). Pathology scores were now elevated in the LPL KO mice when compared to WT littermates (Figure 1B, *p* = 0.054, Mann-Whitney). Sex-based analysis showed that both female and male LPL KO mice were susceptible to keratitis (Supplementary Figure 2).

Because of the similar tendencies for worst disease upon 6294 and PAO1 challenges, all subsequent infection experiments were carried out with *P. aeruginosa* 6294.

**Figure 2:**
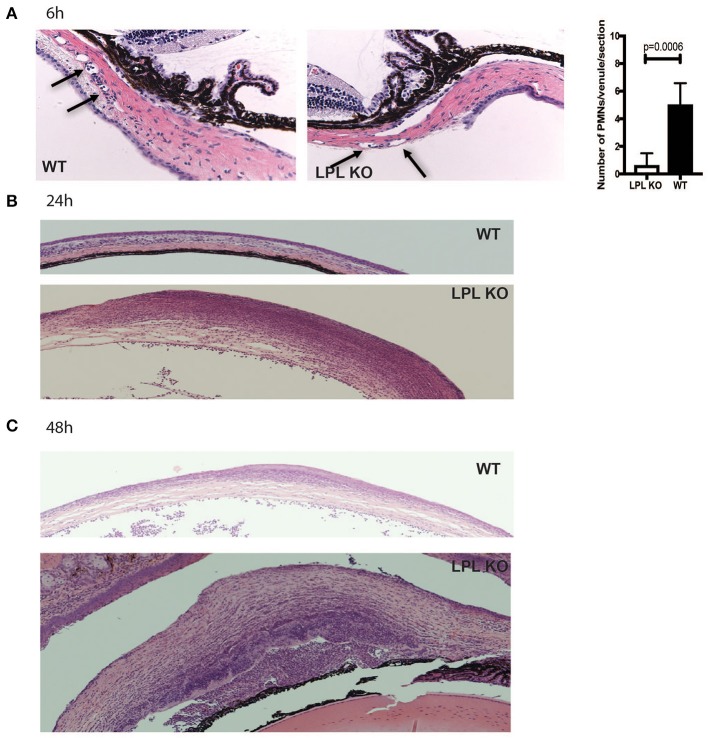
Infected LPL KO mice show altered kinetics of neutrophil recruitment to the infected corneas. Hematoxylin-eosin staining of sections derived from infected LPL-deficient and LPL-sufficient mice. Representative hematoxylin and eosin stained sections form infected with *P. aeruginosa* 6294-infected LPL KO and WT mice at 6 **(A)**, 24 **(B)**, and 48 h **(C)** post-challenge. Arrows point to PMNs present in conjunctival capillaries of the infected animals. Vessel-associated neutrophils were counted in 3 sections per mouse and averaged among 7 animals to gain insight into early neutrophil infiltration. Student's *t*-test. *p* = 0.006. Data are representative images taken from the infected eyes of LPL KO mice (*n* = 5) and LPL WT (*n* = 5) mice. Data demonstrate delays in the trafficking of neutrophils to the infected LPL KO corneas, followed by exacerbated infiltration consistent with worse disease.

To characterize histological changes occurring during *P. aeruginosa* 6294 infection, sections from infected eyes of WT and LPL KO were harvested at 6, 24, and 48 h post-infectious challenge and analyzed for pathophysiological changes (Figure 2). Data revealed significantly fewer neutrophils adhering to the capillaries at 6 h post-challenge (Figure 2, Student's *t*-test, *p* = 0.006), indicative of delayed neutrophil trafficking. This defect was not sustained as there were comparable level of infiltrating neutrophils at 24 h post-infection and significantly more neutrophils in the infected corneas of the LPL KO at 48 h, exemplifying worse disease (Figure 2).

**Figure 3:**
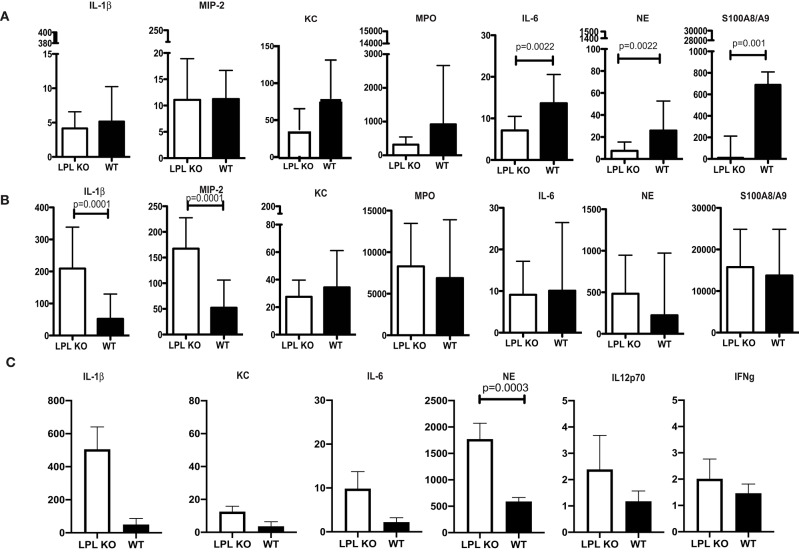
Profiling of corneal inflammatory mediators shows altered inflammatory responses. **(A)** Corneal inflammatory profiles at 6 h post-infection with *P. aeruginosa 6294*. **(B)** Corneal inflammatory profiles at 24 h post-infection. **(C)** Corneal inflammatory profiles at 48 h post-infection. Data plotted in all panels are from groups of 7LPL KO mice and 7 WT littermates mice that were infected with 5 × 10^5^CFU*P. aeruginosa 6294* placed onto scratch-injured eyes. Corneas were harvested either at after infection, washed in F12 media, homogenized in PBS containing a mix of protease inhibitors and supplemented with 0.5% Triton to disrupt plasma membranes. The levels of cytokines in corneal lysates were measured using ELISA, *p*-values by Student's *t*-test. Bonferroni correction for multiple comparisons *p* = 0.007. Data show significant alterations in NE levels indicative of changes in neutrophil trafficking.

Profiling for key infection-associated inflammatory cytokines was carried out at different time points post *P. aeruginosa* 6294 challenge. At 6h after the infectious challenge, no differences in the tissue levels of IL-1βand MIP-2 were observed in corneal lysates (Figure 3). In contrast, KC, MPO, NE, S100A8/9, and IL-6 levels were significantly lower in the infected LPL KO corneas when compared to the WT corneas (Figure 3A, Student's *t*-test). At 24 h post infectious challenge, the neutrophil markers MPO, NE, S100A8/9 were comparable between the infected L-plastin and WT mice. Differences were now observed in the neutrophil recruiting cytokines such as IL-1β and MIP2 (Figure 3, Student's *t*-test, *p* = 0.0001). At 48 h post-challenge, corneal NE levels were more than 3-fold higher than those in the WT controls (Figure 3C, Student's *t*-test, *p* = 0.0003). Overall, there was a good correlation between the histology data and the tissue markers of infection demonstrating early delay in PMN recruitment, followed by extensive PMN presence. Cumulatively, data revealed alterations in neutrophil frequencies and, likely, functionalities been the source for the increased susceptibility to infection.

### L-Plastin Deficiency in the Myeloid Compartment Is Responsible for the Elevated Susceptibility to Infection

**Figure 4:**
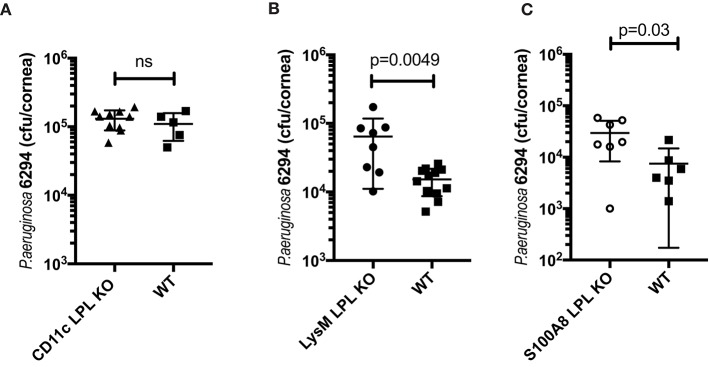
The susceptibility to infection segregated with LysM-driven and S100A8-driven ablation of L-plastin, but not with CD11c-driven ablation. **(A)** LPL deficiency in the CD11c^+^ DCs does not contribute to the phenotype. LPL floxed mice were crossed onto CD11c.Cre^pos^ to generate L-plastin deficiency in the CD11c-expressing cells. CD11c.Cre^pos^LPL ^fl/fl^ mice did not show altered susceptibility to *P. aeruginosa* 6294-induced infection as demonstrated by similar corneal bacterial burden at 24 h post-challenge. Data are from experiments performed in duplicates with each data point representing an individual animal (Student's *t-*test, ns). **(B)** LPL deficiency in the macrophage/neutrophil lineages contributes to susceptibility to disease. LPL floxed mice were crossed onto LysM.Cre^pos^ background to generate L-plastin deficiency in the myeloid/neutrophil lineage. Student's *t*-test, *p* = 0.0049. **(C)** LPL deficiency in the neutrophil lineage contributes to susceptibility to disease. LPL floxed mice were crossed onto S100A8.Cre^pos^ background to generate L-plastin deficiency in the neutrophil lineage. Student's *t*-test, *p* = 0.03. Data prove that L-plastin deficiency in the myeloid compartment determines susceptibility to keratitis.

To examine the impact of L-plastin deficiency in the different myeloid lineages, mice carrying the floxed L-plastin alleles were intercrossed with *CD11c.Cre*^*pos*^, *LysM.Cre*^*pos*^, and *S100A8.Cre*^*pos*^ mice to generate lineage-specific deficiencies. The *CD11c.Cre*^*pos*^*-LPL*^*fl*/*fl*^ mice showed no differential susceptibility to keratitis when compared to WT littermates at either 6 h (data not shown) or 24 h post-infection (Figure 4A, Student's *t*-test, not significant). In contrast, disease susceptibility segregated with LysM-driven ablation of L-plastin (Figure 4B, Student's *t*-test, *p* = 0.0049). The *LysMCre*^*pos*^*LPL*^*flp*/*flp*^ mice showed about 1-log higher recoverable CFU than their infected littermates. Interestingly, the S100A8-driven ablation of L-plastin expression resulted in a weaker, but a significant phenotype, e.g., the bacterial burden in these mice was two-fold higher than that in the WT littermates (Figure 4C, Student's *t*-test, *p* = 0.003). Cumulatively, these data revealed that the L-plastin deficiency in neutrophils sensitized to infection.

### The Elevated Susceptibility to Infection Correlates With Conjunctival Commensal Overgrowth

**Figure 5:**
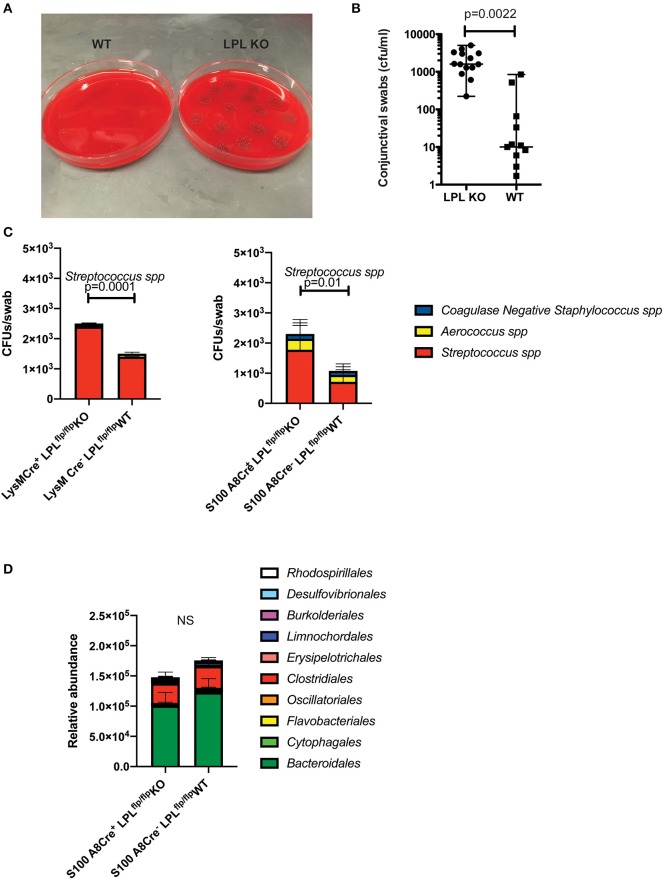
LPL deficient mice show altered conjunctival commensal presence. **(A)** Conjunctival swabs were taken from cohorts of LPL KO (*n* = 7) mice and LPL WT littermates (*n* = 7) and commensal presence identified using LC-MS/MS and quantified by plating on blood agar plates. Representative blood agar plates from LPL WT and LPL KO mice. **(B)** Quantification of the commensal presence per swab. LPL KO mice showed elevated levels of *Streptococcus spp*. when compared to LPL WT mice. Each symbol represents an individual mouse (Mann-Whitney test, *p* = 0.002). **(C)** Conjunctival swabs were taken from cohorts of LysM.Cre^pos^ (*n* = 7) mice, WT littermates and S100A8.Cre^pos^ (*n* = 7) and commensal presence identified using LC-MS/MS and quantified by plating on blood agar plates. Commensal presence was monitored at 7 weeks of age. A statistically significant elevation was detected when the levels of the *Streptococcus spp*. were compared (Two-way ANOVA, *p* = 0.0001 and 0.01, respectively). Experiments were repeated twice with representative data shown. **(D)** Gut commensal abundance was evaluated via 16S metagenomics analysis. Fecal samples were collected from S100A8.Cre^pos^ (*n* = 7) and WT (*n* = 7) littermates, genomic DNA extracted, microbial abundance evaluated (Two-way ANOVA). No significant changes were observed at the order levels. Cumulatively, data demonstrates commensal overgrowth in the absence of L-plastin, a phenotype that appears specific to the ocular niche.

To examine whether the elevated susceptibility to infection correlated with baseline alterations in the commensal presence, conjunctival swabs were collected and commensal bacteria identified. LPL KO mice had abundant incidence of *S. ovis* whereas much fewer bacteria were detected in the WT littermates (Figures 5A,B, Student's *t*-test). Upon microbiolgical analysis, *S. ovis* appeared as a gram-positive, ovoid in shape, catalase negative α-hemolytic strain (data not shown). These experiments were carried out in large cohorts of mice (*N* = 15) where mice were housed in different cages to rule out housing artifacts.

Similar to the LPL KO mice, the *LysM.Cre*^*pos*^*LPL*^*fl*/*fl*^ and *S100A8.Cre*^*pos*^*LPL*^*fl*/*fl*^ mice had increased abundance of *Streptococcal spp*. (Figure 5C, Two-way ANOVA, *p* = 0.0001 an *p* = 0.01, respectively). The commensal overgrowth was ocular-niche specific, as metagenomics 16S analysis did not reveal significant alterations in the gut commensal abundance at the order, family or individual strain levels (Figure 5D, Two-way ANOVA). Cumulatively, data demonstrate that defects in neutrophil functionality correlate with ocular, but not gut commensal overgrowth.

### L-Plastin Deficient Mice Show Reduced Neutrophil Presence and Compromised Neutrophil Functions

**Figure 6:**
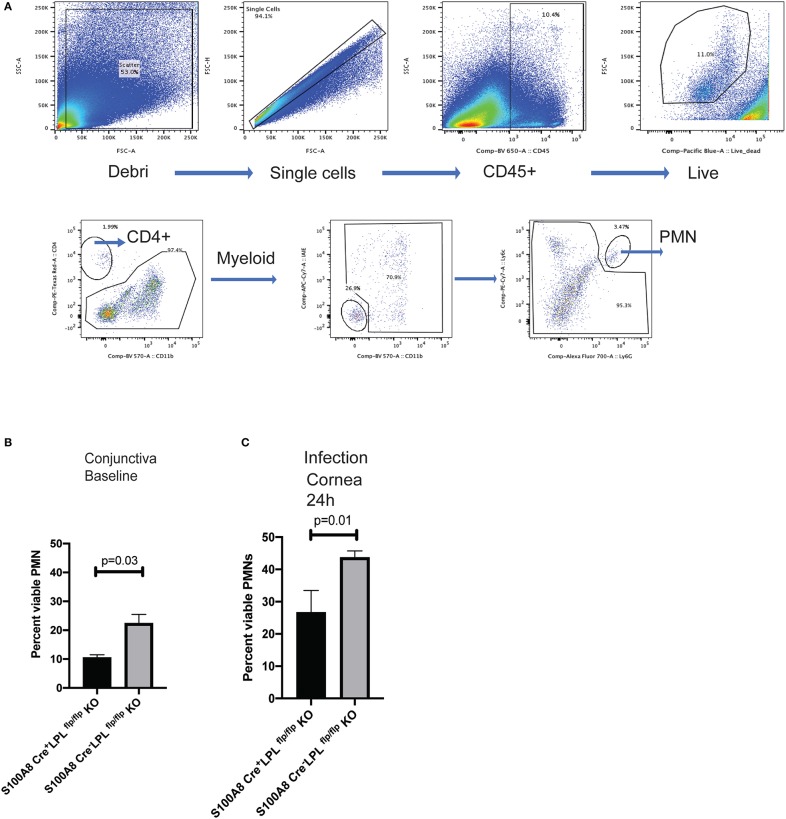
S100A8.Cre^pos^mice show diminished frequencies of viable PMNs at baseline and during infection. Gating strategy for analysis of the conjunctival tissues harvested from naïve S100A8.Cre^pos^and S100A8.Cre^neg^mice. Neutrophils were identified as CD45^+^, viable, Ly6G^+^ cells. Flow cytometry analysis using CD45, CD11b, Ly6G was carried out on pooled conjunctival and corneal tissues from non-infected LPL KO and WT littermates. **(A)** Percent CD45^+^, viable, Ly6G^+^ cells in the conjunctival tissues. At least 5 mice per genotype were harvested and analyzed individually. Experiments were performed twice with similar outcomes (Student's *t*-test, *p* = 0.03). **(B)** Percent CD45^+^, viable, Ly6G^+^ cells in the infected corneas. Mice were infected with 5 × 10^5^ CFU *P. aeruginosa* 6294 per eye and corneal tissues harvested at 24 h post-challenge. At least 5 mice per genotype were analyzed individually. Experiments were performed twice with similar outcomes (Student's *t*-test, *p* = 0.01). Cumulatively data demonstrate significant reduction in the viable PMN frequencies at baseline and during infection in the L-plastin deficient KO mice.

To examine lymphocyte distribution in naïve, non-infected animals, conjunctival tissues were harvested and the frequencies of CD4+ T cells, DCs, myeloid cells and PMNs quantified in the *S100A8.Cre*^*neg*^*LPL*^*fl*/*fl*^ *and S100A8.Cre*^*pos*^*LPL*^*fl*/*fl*^ mice. The most striking differences were observed in the PMN populations, with the relative percent of the viable PMNs been significantly higher in the *S100A8.Cre*^*neg*^*LPL*^*fl*/*fl*^ mice, WT, littermates when compared to *S100A8.Cre*^*pos*^*LPL*^*fl*/*fl*^ mice KO mice (Figures 6A,B, Student's *t*-test, *p* = 0.03). Next, we examined the relative abundance of viable PMNs in the corneas of *P. aeruginosa*-infected mice (Figure 6C). At 24 h post-infectious challenge, a time point at which bacterial burdens in the cornea were different among the genotypes, the numbers of viable PMNs were significantly reduced in the *S100A8.Cre*^*pos*^*LPL*^*fl*/*fl*^ mice when compared to the L-plastin sufficient mice (Figure 6C, Student's *t-*test, *p* = 0.01).

**Figure 7:**
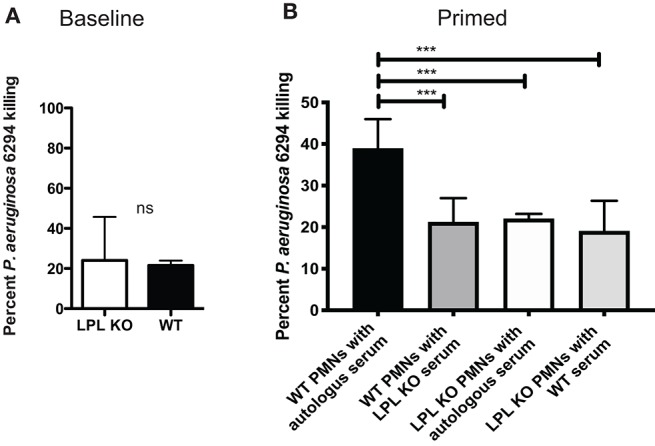
L-plastin deficient PMNs show diminished bactericidal activities. **(A)** LPL-deficient and sufficient neutrophils were purified from bone marrow and exposed to *P. aeruginosa* at MOI of 0.05. No differences in the bactericidal activities were noted when the reactions were conducted in the presence of autologous serum. **(B)** LPL-deficient show compromised bactericidal activity against *P. aeruginosa*. OPK assays were carried out with bone marrow derived neutrophils harvested from infected mice in the presence of either autologous serum harvested from WT mice or serum harvested from infected LPL KO mice. Percent killed *P. aeruginosa* was plotted (One-way ANOVA, overall *p* = 0.001). Significant differences were denoted by asterisks. Data are representative of duplicate experiments carried out with at least 4 biological replicas. Cumulatively data demonstrate significant reduction in bactericidal activities upon exposure to signals derived from “infected” serum.

To evaluate whether changes in bactericidal activities existed in the absence of L-plastin, bone marrow-derived neutrophils were purified from naïve or infected mice. There were no significant differences in the bactericidal potential of non-primed neutrophils (Figure 7A). In contrast, upon infection, there were measurable changes in the bactericidal potency of neutrophils. WT neutrophils showed improved killing, whereas L-plastin deficient neutrophils failed to respond to priming signals (Figure 7B, One-way ANOVA, overall *p* = 0.0001).

**Figure 8:**
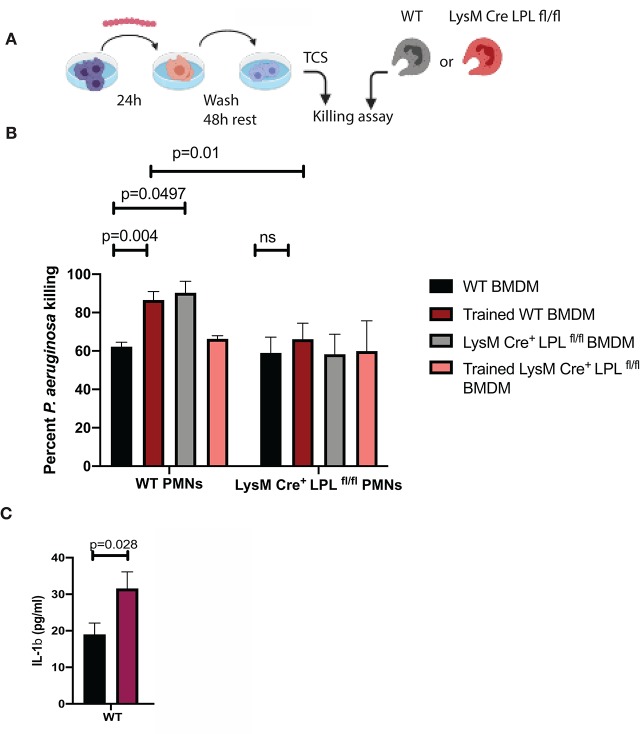
LPL deficient neutrophils show compromised responses to priming signals, released by “trained” macrophages. **(A)** Schematic diagram depicting the experimental approach to generate “trained” macrophages. BMDMs were exposed to *S. ovis* (MOI = 1) for 24 h, cells were washed, rested for 48 h, then conditioned supernatants were harvested, and used to prime neutrophil killing. **(B)** Killing assays were carried out with WT PMNs (first set of bars) and *LysMCre*^*pos*^*LPL*^*fl*.*fl*^ PMNs (second set of bars) in the presence of conditioned medium derived from “non-trained” WT BMDMs (black bars), “trained” WT BMDMs (red bars), “non-trained” *LysMCre*^*pos*^*LPL*^*fl*.*fl*^ BMDMs (gray bars) and “trained” *LysMCre*^*pos*^*LPL*^*fl*.*fl*^ BMDMs (pink bars) (Two-way ANOVA, *p* < 0.05). Data are representative of two experiments. Each bar shows mean values with SD based on four individual replicas per sample. **(C)** ELISA measurements of IL-1β in the conditioned media from “non-trained” WT (black bars) and “trained” (purple bars) BMDMs. (Student's *t*-test, *p* < 0.028). Representative data are shown out of two experiments. Cumulatively, data demonstrate that exposure to commensals “train” macrophages to prime neutrophil responses to *P. aeruginosa*. The *LysMCre*^*pos*^*LPL*^*fl*.*fl*^ PMNs show bactericidal defects revealed upon exposure to priming signals.

Since macrophages produce pro-inflammatory factors that control neutrophil infiltration and activation during keratitis (18–20), we examined whether conditioned media derived from *in vitro* cultured BMDM could affect PMN bactericidal activities in a similar fashion like serum. L-plastin sufficient BMDMs were cultured either exposed to the commensal isolate “trained” or left untreated, “non-trained,” washed, rested for 48 h, and conditioned media was collected (Figure 8A). Under non-activated conditions, there were no major differences in the bactericidal activities of L-plastin sufficient (first set of bars, black bar, Figure 8B) and L-plastin deficient (second set of bars, black bar, Figure 8B). In contrast, conditioned media from commensal-exposed, “trained” WT BMDMs promoted neutrophil bactericidal activities in WT PMNs (Figure 8B, black vs. red bars, Two-way ANOVA, *p* = 0.004). The *LysM.Cre*^*pos*^ *LPL*^*fl*/*fl*^ PMNs did not respond to the priming signals by the conditioned media (Figure 8B, second set of bars, Two-way ANOVA, not significant). Cumulatively, these data confirm that defects in neutrophil functionalities could be revealed upon priming.

## Discussion

The ocular mucosal site is paucibacterial, in stark contrast to the skin or other mucosal sites such as the gut. The types of cultivable bacterial species range from none to 2–3 per eye per sampling (21, 22). This incredibly low bacterial presence is an unique feature of the site. Several different mechanisms have been proposed including tear film, mucins, antimicrobial proteins, sIgA, frequent blinking (22–24). Our previous work has also shown that neutrophils can be recruited to the conjunctival tissues in response to commensal presence (11, 25). Here, we define an important role for L-plastin in the myeloid compartment and, specifically, in neutrophils that controls commensal burden and susceptibility to infection.

The absence of L-plastin resulted in ocular commensal conjunctival overgrowth. LPL KO mice showed increased *Streptococcus ovis* burden and a similar phenotype was observed in mice that harbored lineage-specific deletion of L-plastin in the myeloid or neutrophil compartments such as *LysMCre*^*pos*^*LPL*^*fl*.*fl*^ and *S100A8.Cre*^*pos*^*LPL*^*fl*/*fl*^ mice indicating that the neutrophil responses regulated by L-plastin were fundamental to control commensal abundance. While other bacterial species such as *CNS spp*. and *Aerococcus spp*. were also isolated from the *LysMCre*^*pos*^*LPL*^*fl*.*fl*^ and *S100A8.Cre*^*pos*^*LPL*^*fl*/*fl*^ mice, their abundances were not different among the genotypes, suggesting that the responses were organism-specific. The elevated levels of *Streptococcus* spp. correlated with reduced levels of myeloid cells measured in L-plastin deficient mice at steady state (data not shown). Indeed, subsequent experiments showed that neutrophil frequencies were significantly reduced in the *S100A8.Cre*^*pos*^*LPL*^*fl*/*fl*^ conjunctivas when compared to WT tissues, suggesting that changes in neutrophil trafficking likely affect commensal presence. Cumulatively, data illustrate niche-and organism-specific mechanisms to control ocular commensal presence.

In addition to alterations in the commensal presence, we observed elevated susceptibility to *P. aeruginosa*-induced keratitis. The susceptibility trait segregated with the L-plastin defects in the myeloid compartment and neutrophils, but not in the CD11c+ cells. Mice with lineage-specific deletions of L-plastin under the *LysMCre*^*pos*^ and *S100A8.Cre*^*pos*^ promoters showed elevated bacterial burden in the eye, reduced neutrophil frequencies, and impaired neutrophil bactericidal activities. Given that L-plastin transmits integrin mediated adhesion, it wasn't surprising to detect reductions in PMN levels at baseline and upon challenge (10). However, similar analysis of the lungs of non-infected mice or *Streptococcus pneumoniae*-infected mice failed to show differences in viable PMN counts, suggesting that the observed phenotype is organ-specific (7). It is likely that the decreased PMN levels were reflective of altered trafficking, rather than viability, as no changes in the numbers of non-viable PMNs were noted (data not shown). We also detected reductions in the bactericidal capacity in the L-plastin deficient PMNs, which were observed only after exposure to serum-derived priming signals. Similar phenotype was noted, when L-plastin deficient PMNs were allowed to opsonophagocytose bacteria in the presence of conditioned medium derived from BMDMs.

Macrophages are the most abundant, long-lived myeloid cell in the conjunctival and corneal tissues. Their frequency and functionalities depend on microbiota (26). Recently, it was shown that macrophages retain innate memory. Exposure to commensal metabolites such as β-glucans or BCG trains macrophage responses through epigenetic modifications to subsequent challenges (27–30). The training is usually associated with a shift in the metabolic responses or cytokine release (31, 32). How commensal exposure affects *P. aeruginosa* macrophage responsiveness hasn't been investigated. Prior exposure to commensal organisms *in vitro* affected subsequent responses to *P. aeruginosa* challenge in, both, BMDMs themselves and PMNs. Namely, BMDMs exposed to *Streptococcus* spp. released elevated levels of IL-1β upon secondary challenge with *P. aeruginosa* (Figure 8C) and their bactericidal function was improved in contrast to that of the L-plastin deficient BMDM (Supplementary Figure 3). Similar to the “trained” macrophages, L-plastin sufficient PMNs demonstrated improved bactericidal activity in the presence of conditioned medium derived from “trained” BMDMs, in contrast to the phenotype of the L-plastin deficient PMNs. It is likely that IL-1β, released by the BMDMs promotes neutrophil priming.

Microbiota-driven changes of the ocular mucosa are often associated with alterations in the IL-1β levels (11, 25, 33). For example, GF out bred Swiss Webster (SW) mice showed significantly lower IL-1β levels when compared to Specific Pathogen Free (SPF) mice illustrating that IL-1β signals were induced by commensal exposure (11). Consistently, increased IL-1β production was elicited upon *Corynebacterium mastitidis* colonization of ocular conjunctiva (25). In contrast, deficiency in the IL-1β signaling such as in the IL-1R knock-out mice was linked to increased abundance of commensal species in the cornea including *Streptococcus spp*., demonstrating that IL-1β signals restricted ocular commensal presence (34). What remains unknown to a large extent is what cell types respond to IL-1β and whether these responses are niche-specific. Microbiota-induced IL-1β responses in the gut stimulate protective monocytic activation, while microbiota-induced IL-1β responses in the skin stimulate T_C_17 cell responses (35, 36). Consistently, our data provides information that trained macrophages release elevated levels of IL-1β upon priming and that LPL deficient PMNs mount decreased ROS production and impaired *P. aeruginosa* killing after IL-1β priming (data not shown) (37).

In conclusion, we provide evidence of defective neutrophil functionality in the absence of L-plastin that is associated with commensal overgrowth coupled to increased sensitivity to opportunistic infections. Our work has important implications, suggesting that genetic predispositions associated with commensal overgrowth can be associated with frequent opportunistic infections.

## Data Availability Statement

The datasets generated for this study can be found in figshare at https://figshare.com/s/eb65063866dd2b4e0fee.

## Ethics Statement

The animal study was reviewed and approved by BWH IACUC.

## Author Contributions

XL, AK, KS-P, YR, JL, TL, SKM, and SL performed experiments, analyzed data, and read the manuscript. SCM provided mice and read the manuscript. RF, SC, and DS suggested experiments and read the manuscript. MG performed experiments, analyzed data, conceptualized the study, and wrote the manuscript.

### Conflict of Interest

The authors declare that the research was conducted in the absence of any commercial or financial relationships that could be construed as a potential conflict of interest.

## Supplementary Material

Supplementary Figure 1 L-plastin deficiency sensitizes to *P. aeruginosa*-induced keratitis. **(A)** Bacterial burdens and pathology scores at 24 h post infection. Groups of LPL KO mice (*n* = 7) and WT littermates (*n* = 14) mice were infected with 5 × 10^6^ CFU *P. aeruginosa* PAO1 per eye. LPL deficient mice have higher bacterial burden than WT littermates at 24 h after the PAO1 challenge. Data are representative of two independent experiments performed under comparable conditions. In the scatter plot each symbol represents CFU value per individual animal. *p*-values are generated using Student's *t*-test, *p* < 0.0001. Box with whiskers show pathology scores, *p*-values are generated using Mann Whitney test, *p* = 0.0062. **(B)** Bacterial burdens at 48 h post infection with 5 × 10^6^ CFU *P. aeruginosa* PAO1. Groups of LPL KO mice (*n* = 5) and WT littermates (*n* = 5) mice were infected with *P. aeruginosa* PAOI per eye. Data are representative of two independent experiments performed under comparable conditions. *p*-values are generated using Student's *t*-test, *p* = 0.03. Box with whiskers show pathology scores, *p*-values are generated using Mann Whitney test, *p* = 0.0054.

Supplementary Figure 2 Male and female LPL mice show comparable susceptibility to infection. Groups of LPL KO male mice (*n* = 7), female LPL KO mice (*n* = 5), and age and gender matching littermates (*n* = 7) mice were infected with 5 × 10^5^ CFU *P. aeruginosa* 6294 per eye. Data are representative of three independent experiments performed under comparable conditions. *p*-values are generated using Mann-Whitney test. Cumulatively, these data show no sex influence on the phenotype. ^*^*p* < 0.05; ^**^*p* < 0.001; ^***^*p* < 0.0001.

Supplementary Figure 3 Exposure to *Streptococcus spp*. promotes BMDM bactericidal activity. BMDMs were exposed to *S. ovis* (MOI = 1) for 24 h, cells were washed, rested for 48 h, then exposed to *P. aeruginosa* 6294 MOI 1 for 60 min. Cells were treated with gentamycin for 90 min and then lysed to count viable intracellular bacteria. Bars represent mean cfu values with SD. Each symbol is a biological replica. One-way AOVA, *p* = 0.072.

## References

[1] Shinomiya H Hirata H Saito S Yagisawa H Nakano M. Identification of the 65-kDa phosphoprotein in murine macrophages as a novel protein: homology with human L-plastin. Biochem Biophys Res Commun. (1994) 202:1631–8. 10.1006/bbrc.1994.2120 8060349

[2] Shinomiya H. Plastin family of actin-bundling proteins: its functions in leukocytes, neurons, intestines, and cancer. Int J Cell Biol. (2012) 2012:213492. 10.1155/2012/213492 22262972 PMC3259490

[3] Kell MJ Riccio RE Baumgartner EA Compton ZJ Pecorin PJ Mitchell TA. Targeted deletion of the zebrafish actin-bundling protein L-plastin (lcp1). PLoS ONE. (2018) 13:e0190353. 10.1371/journal.pone.0190353 29293625 PMC5749806

[4] Morley SC. The actin-bundling protein L-plastin: a critical regulator of immune cell function. Int J Cell Biol. (2012) 2012:935173. 10.1155/2012/935173 22194750 PMC3238366

[5] Morley SC. The actin-bundling protein L-plastin supports T-cell motility and activation. Immunol Rev. (2013) 256:48–62. 10.1111/imr.12102 24117812 PMC3801223

[6] Todd EM Deady LE Morley SC. Intrinsic T- and B-cell defects impair T-cell-dependent antibody responses in mice lacking the actin-bundling protein L-plastin. Eur J Immunol. (2013) 43:1735–44. 10.1002/eji.201242780 23589339 PMC3794664

[7] Deady LE Todd EM Davis CG Zhou JY Topcagic N Edelson BT. L-plastin is essential for alveolar macrophage production and control of pulmonary pneumococcal infection. Infect Immun. (2014) 82:1982–93. 10.1128/IAI.01199-13 24595139 PMC3993441

[8] Todd EM Zhou JY Szasz TP Deady LE D'Angelo JA Cheung MD. Alveolar macrophage development in mice requires L-plastin for cellular localization in alveoli. Blood. (2016) 128:2785–96. 10.1182/blood-2016-03-705962 27758872 PMC5159703

[9] Dubey M Singh AK Awasthi D Nagarkoti S Kumar S Ali W. L-Plastin S-glutathionylation promotes reduced binding to beta-actin and affects neutrophil functions. Free Radic Biol Med. (2015) 86:1–15. 10.1016/j.freeradbiomed.2015.04.008 25881549

[10] Chen H Mocsai A Zhang H Ding RX Morisaki JH White M. Role for plastin in host defense distinguishes integrin signaling from cell adhesion and spreading. Immunity. (2003) 19:95–104. 10.1016/S1074-7613(03)00172-9 12871642

[11] Kugadas A Christiansen SH Sankaranarayanan S Surana NK Gauguet S Kunz R. Impact of microbiota on resistance to ocular *Pseudomonas aeruginosa*-induced keratitis. PLoS Pathog. (2016) 12:e1005855. 10.1371/journal.ppat.1005855 27658245 PMC5033354

[12] Morley SC Wan C Lo WL Lio CW Zinselmeyer BH Miller MJ. The actin-bundling protein L-plastin dissociates CCR7 proximal signaling from CCR7-induced motility. J Immunol. (2010) 184:3628–8. 10.4049/jimmunol.0903851 20194718 PMC2855624

[13] Stranges PB Watson J Cooper CJ Choisy-Rossi CM Stonebraker AC Beighton RA. Elimination of antigen-presenting cells and autoreactive T cells by Fas contributes to prevention of autoimmunity. Immunity. (2007) 26:629–41. 10.1016/j.immuni.2007.03.016 17509906 PMC2575811

[14] Preston MJ Fleiszi SM Zaidi TS Goldberg JB Shortridge VD Vasil ML. Rapid and sensitive method for evaluating *Pseudomonas aeruginosa* virulence factors during corneal infections in mice. Infect Immun. (1995) 63:3497–501. 10.1128/IAI.63.9.3497-3501.1995 7642283 PMC173483

[15] Gadjeva M Wang Y Horwitz BH. NF-kappaB p50 and p65 subunits control intestinal homeostasis. Eur J Immunol. (2007) 37:2509–17. 10.1002/eji.200737186 17705134

[16] Khandelwal P Blanco-Mezquita T Emami P Lee HS Reyes NJ Mathew R. Ocular mucosal CD11b+ and CD103+ mouse dendritic cells under normal conditions and in allergic immune responses. PLoS ONE. (2013) 8:e64193. 10.1371/journal.pone.0064193 23691170 PMC3653857

[17] Dwyer M Gadjeva M. Opsonophagocytic assay. Methods Mol Biol. (2014) 1100:373–9. 10.1007/978-1-62703-724-2_32 24218277

[18] Karmakar M Sun Y Hise AG Rietsch A Pearlman E. Cutting edge: IL-1beta processing during Pseudomonas aeruginosa infection is mediated by neutrophil serine proteases and is independent of NLRC4 and caspase-1. J Immunol. (2012) 189:4231–5. 10.4049/jimmunol.1201447 23024281 PMC3482477

[19] Rudner XL Kernacki KA Barrett RP Hazlett LD. Prolonged elevation of IL-1 in *Pseudomonas aeruginosa* ocular infection regulates macrophage-inflammatory protein-2 production, polymorphonuclear neutrophil persistence, and corneal perforation. J Immunol. (2000) 164:6576–82. 10.4049/jimmunol.164.12.6576 10843717

[20] Thakur A Barrett RP McClellan S Hazlett LD. Regulation of *Pseudomonas aeruginosa* corneal infection in IL-1 beta converting enzyme (ICE, caspase-1) deficient mice. Curr Eye Res. (2004) 29:225–33. 10.1080/02713680490516710 15590467

[21] Kugadas A Gadjeva M. Impact of microbiome on ocular health. Ocul Surf. (2016) 14:342–9. 10.1016/j.jtos.2016.04.004 27189865 PMC5082109

[22] Cavuoto KM Banerjee S Galor A. Relationship between the microbiome and ocular health. Ocul Surf. (2019) 17:384–92. 10.1016/j.jtos.2019.05.006 31125783

[23] McDermott AM. Antimicrobial compounds in tears. Exp Eye Res. (2013) 117:53–61. 10.1016/j.exer.2013.07.014 23880529 PMC3844110

[24] Qin G Baidouri H Glasser A Raghunathan V Morris C Maltseva I. Development of an in vitro model to study the biological effects of blinking. Ocul Surf. (2018) 16:226–34. 10.1016/j.jtos.2017.12.002 29309844

[25] St Leger AJ Desai JV Drummond RA Kugadas A Almaghrabi F Silver P. An ocular commensal protects against corneal infection by driving an interleukin-17 response from mucosal gammadelta T cells. Immunity. (2017) 47:148–58 e145. 10.1016/j.immuni.2017.06.014 28709803 PMC5553552

[26] Wu M Liu J Li F Huang S He J Xue Y. Antibiotic-induced dysbiosis of gut microbiota impairs corneal development in postnatal mice by affecting CCR2 negative macrophage distribution. Mucosal Immunol. (2019) 13:47–63. 10.1038/s41385-019-0193-x 31434991 PMC6914671

[27] Netea MG Joosten LA Latz E Mills KH Natoli G Stunnenberg HG. Trained immunity: a program of innate immune memory in health and disease. Science. (2016) 352:aaf1098. 10.1126/science.aaf1098 27102489 PMC5087274

[28] Netea MG Quintin J van der Meer JW. Trained immunity: a memory for innate host defense. Cell Host Microbe. (2011) 9:355–61. 10.1016/j.chom.2011.04.006 21575907

[29] Crisan TO Netea MG Joosten LA. Innate immune memory: implications for host responses to damage-associated molecular patterns. Eur J Immunol. (2016) 46:817–28. 10.1002/eji.201545497 26970440

[30] Arts RJW Carvalho A La Rocca C Palma C Rodrigues F Silvestre R. Immunometabolic pathways in BCG-induced trained immunity. Cell Rep. (2016) 17:2562–71. 10.1016/j.celrep.2016.11.011 27926861 PMC5177620

[31] Saeed S Quintin J Kerstens HH Rao NA Aghajanirefah A Matarese F. Epigenetic programming of monocyte-to-macrophage differentiation and trained innate immunity. Science. (2014) 345:1251086. 10.1126/science.1251086 25258085 PMC4242194

[32] Cheng SC Quintin J Cramer RA Shepardson KM Saeed S Kumar V. mTOR- and HIF-1alpha-mediated aerobic glycolysis as metabolic basis for trained immunity. Science. (2014) 345:1250684. 10.1126/science.1250684 25258083 PMC4226238

[33] Sahin A Yildirim N Gultekin S Akgun Y Kiremitci A Schicht M. Changes in the conjunctival bacterial flora of patients hospitalized in an intensive care unit. Arq Bras Oftalmol. (2017) 80:21–4. 10.5935/0004-2749.20170007 28380097

[34] Wan SJ Sullivan AB Shieh P Metruccio MME Evans DJ Bertozzi CR. IL-1R and MyD88 contribute to the absence of a bacterial microbiome on the healthy murine cornea. Front Microbiol. (2018) 9:1117. 10.3389/fmicb.2018.01117 29896179 PMC5986933

[35] Seo SU Kamada N Munoz-Planillo R Kim YG Kim D Koizumi Y. Distinct commensals induce interleukin-1beta via NLRP3 inflammasome in inflammatory monocytes to promote intestinal inflammation in response to injury. Immunity. (2015) 42:744–55. 10.1016/j.immuni.2015.03.004 25862092 PMC4408263

[36] Paller AS Kong HH Seed P Naik S Scharschmidt TC Gallo RL. The microbiome in patients with atopic dermatitis. J Allergy Clin Immunol. (2019) 143:26–35. 10.1016/j.jaci.2018.11.015 30476499 PMC7163929

[37] Van Ziffle JA Lowell CA. Neutrophil-specific deletion of Syk kinase results in reduced host defense to bacterial infection. Blood. (2009) 114:4871–82. 10.1182/blood-2009-05-220806 19797524 PMC2786293

