# Supplementary figures and images for: Neutrophil L-Plastin Controls Ocular Paucibacteriality and Susceptibility to Keratitis
Source: Front Immunol. 2020 Apr 3;11:547. doi: 10.3389/fimmu.2020.00547 (PMC7147296; doi:10.3389/fimmu.2020.00547)

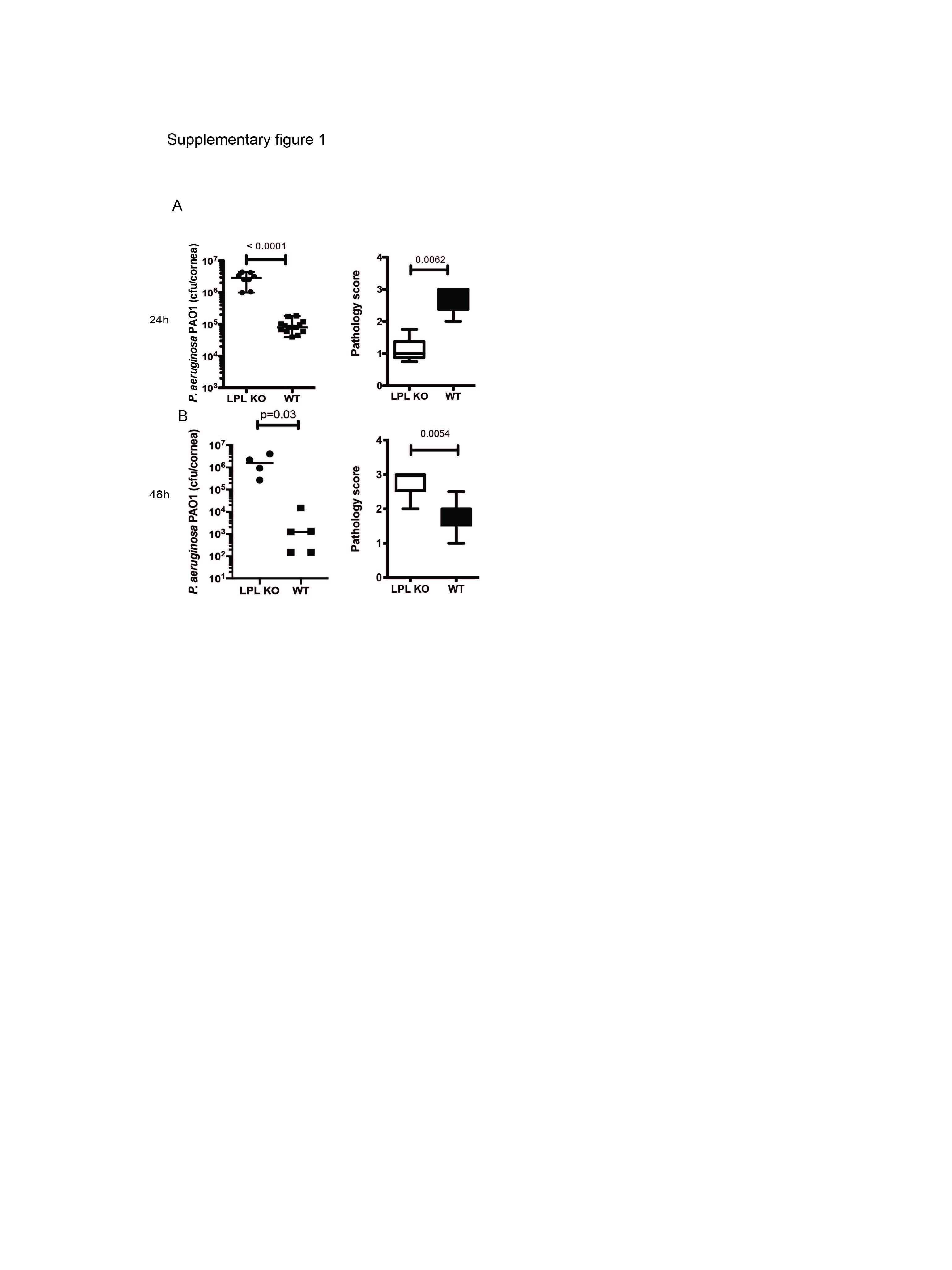

Supplement: Supplementary Figure 1 — L-plastin deficiency sensitizes to P. aeruginosa-induced keratitis. (A) Bacterial burdens and pathology scores at 24 h post infection. Groups of LPL KO mice (n = 7) and WT littermates (n = 14) mice were infected with 5 × 10^6^ CFU P. aeruginosa PAO1 per eye. LPL deficient mice have higher bacterial burden than WT littermates at 24 h after the PAO1 challenge. Data are representative of two independent experiments performed under comparable conditions. In the scatter plot each symbol represents CFU value per individual animal. p-values are generated using Student's t-test, p < 0.0001. Box with whiskers show pathology scores, p-values are generated using Mann Whitney test, p = 0.0062. (B) Bacterial burdens at 48 h post infection with 5 × 10^6^ CFU P. aeruginosa PAO1. Groups of LPL KO mice (n = 5) and WT littermates (n = 5) mice were infected with P. aeruginosa PAOI per eye. Data are representative of two independent experiments performed under comparable conditions. p-values are generated using Student's t-test, p = 0.03. Box with whiskers show pathology scores, p-values are generated using Mann Whitney test, p = 0.0054. [file Image_1.JPEG]

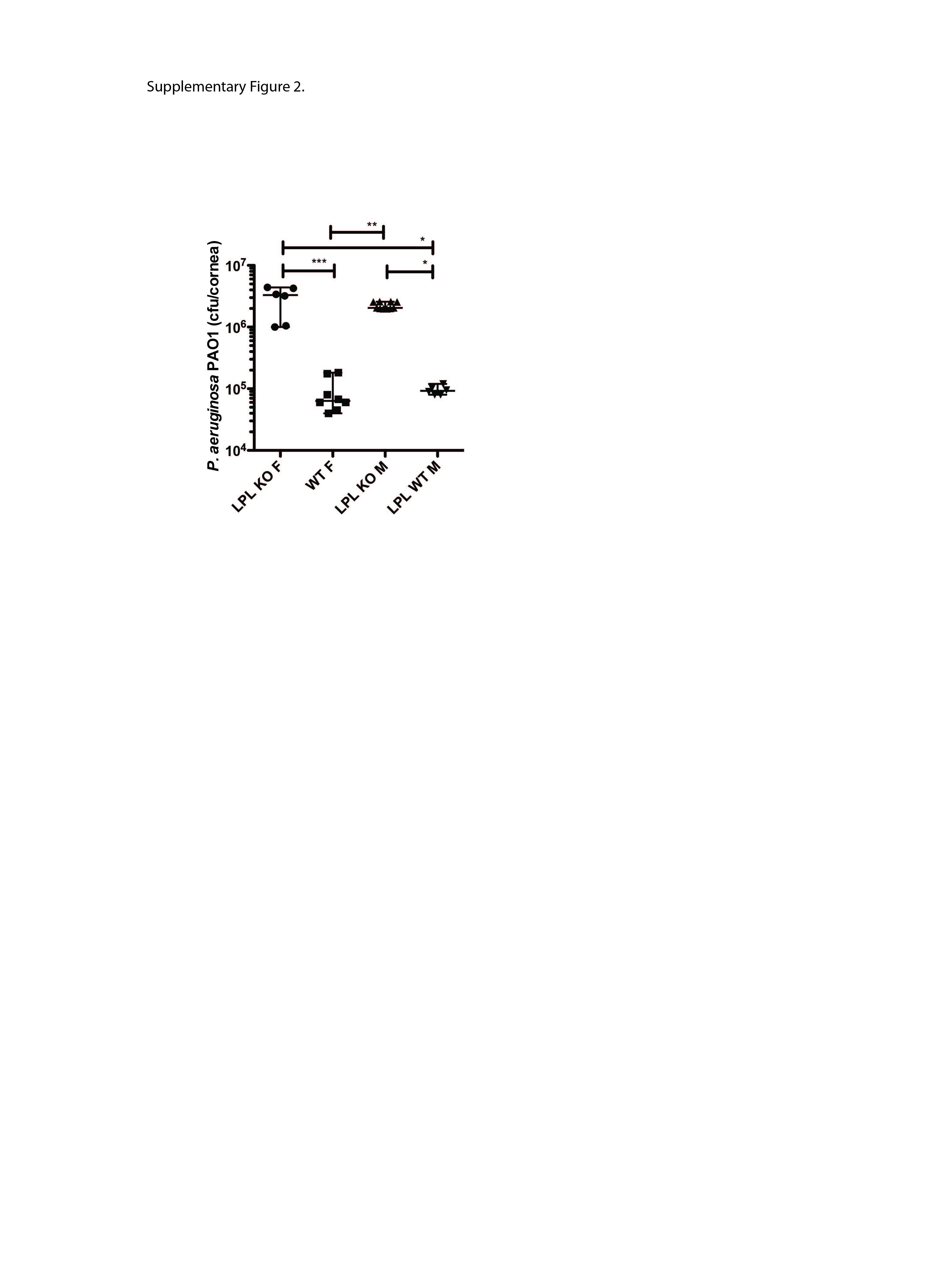

Supplement: Supplementary Figure 2 — Male and female LPL mice show comparable susceptibility to infection. Groups of LPL KO male mice (n = 7), female LPL KO mice (n = 5), and age and gender matching littermates (n = 7) mice were infected with 5 × 10^5^ CFU P. aeruginosa 6294 per eye. Data are representative of three independent experiments performed under comparable conditions. p-values are generated using Mann-Whitney test. Cumulatively, these data show no sex influence on the phenotype. *p < 0.05; **p < 0.001; ***p < 0.0001. [file Image_2.JPEG]

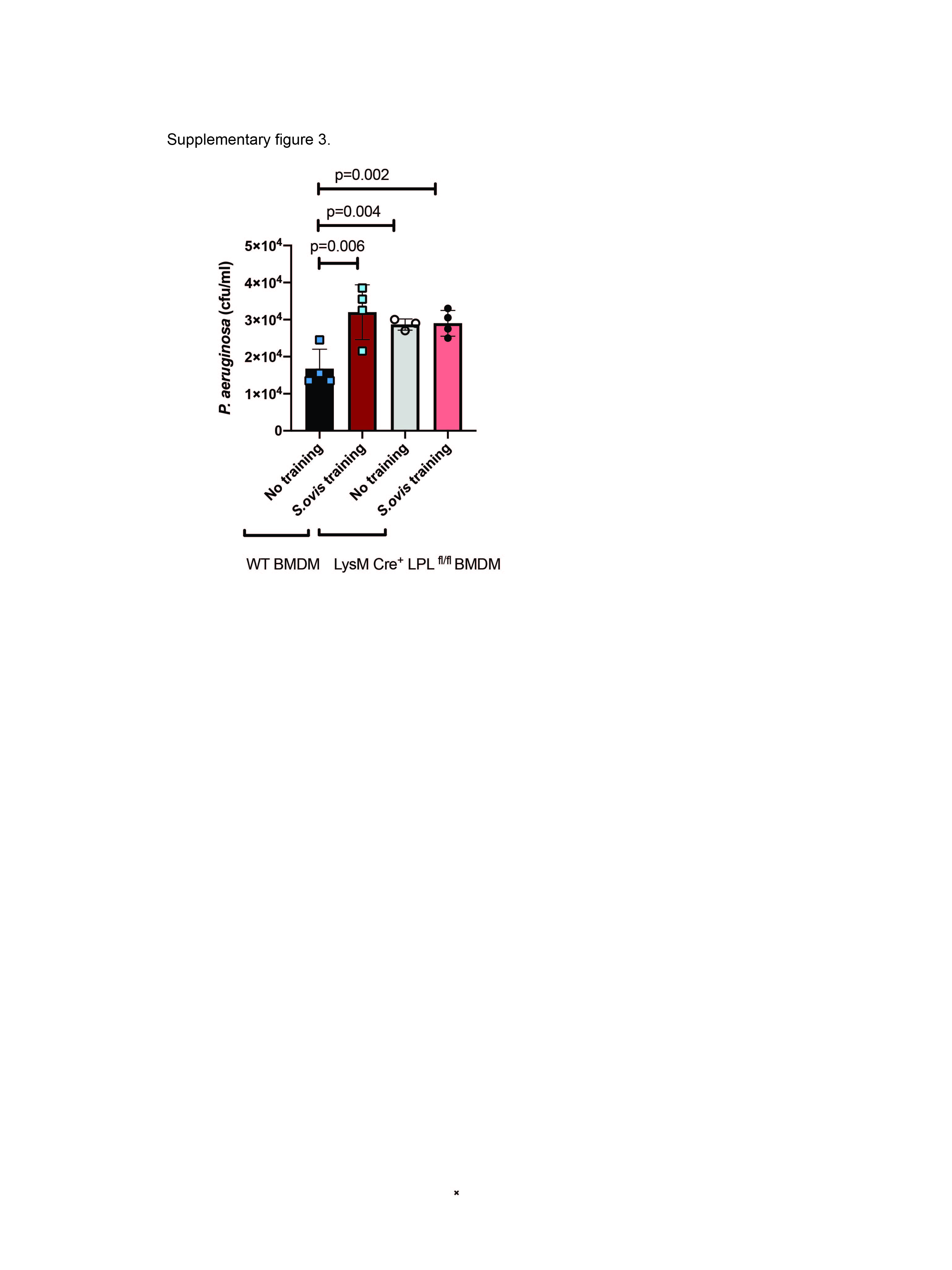

Supplement: Supplementary Figure 3 — Exposure to Streptococcus spp. promotes BMDM bactericidal activity. BMDMs were exposed to S. ovis (MOI = 1) for 24 h, cells were washed, rested for 48 h, then exposed to P. aeruginosa 6294 MOI 1 for 60 min. Cells were treated with gentamycin for 90 min and then lysed to count viable intracellular bacteria. Bars represent mean cfu values with SD. Each symbol is a biological replica. One-way AOVA, p = 0.072. [file Image_3.JPEG]
